# A Case of Autoimmune Pulmonary Alveolar Proteinosis

**DOI:** 10.1002/ccr3.72040

**Published:** 2026-02-17

**Authors:** Meher Binte Ali, Danish Jilani, Syeda Hafsa Qadri, Yilin Song, Nancy Hardy

**Affiliations:** ^1^ University of Maryland Medical Center Baltimore Maryland USA; ^2^ Dow University of Health Sciences Pakistan

**Keywords:** anti‐GM‐CSF, autoimmune, pulmonary alveolar proteinosis, rituximab

## Abstract

Chemotherapeutic agents or regular doses of Rituximab may represent a potential therapeutic option for refractory cases of autoimmune pulmonary alveolar proteinosis.

## Graphical abstract

CT scan showing widespread ground‐glass opacities and thickened septal lines in all five lobes, described as ‘crazy paving pattern’, pathognomonic for pulmonary alveolar proteinosis.

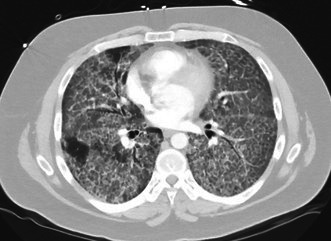

## 1 Introduction

Autoimmune pulmonary alveolar proteinosis (PAP) is a rare restrictive lung disease, characterized by accumulation of surfactant in the lung due to autoantibodies against granulocyte macrophage colony stimulating factor (GM‐CSF) [1]. This causes dysfunctional alveolar macrophages, which are unable to clear the surfactant from the alveoli, resulting in respiratory failure and frequent infections [2]. This condition can be congenital, secondary, or autoimmune. Congenital PAP is caused by genetic mutations in the GM‐CSF receptor. Secondary PAP is due to reduced number of alveolar macrophages due to causes like chronic infections, inflammation, drugs, environmental exposures, and malignancy [3]. Autoimmune PAP, the most common type, is initiated by immunoglobulin‐G anti‐GM‐CSF antibodies, which decrease the number of functional alveolar macrophages [4]. Patients commonly present with dyspnea, cough, weight loss, and fatigue and may have a history of smoking [5]. On examination, cyanosis, clubbing, and inspiratory crackles are commonly seen [3]. Here we present a case of autoimmune PAP refractory to standard therapy.

## 2 Case History/Examination

A 28‐year‐old male with a history of recurrent bronchitis as a child presented with progressively worsening shortness of breath for the past 3 years. He also reported a 60‐pound weight loss during that time. He was evaluated at various urgent cares for progressive dyspnea and completed a 3‐month course of corticosteroids without any benefit. He was on 6 L oxygen at rest which limited his time outside the house. He came for a second opinion to our clinic. On examination, he had bilateral inspiratory crackles and decreased breath sounds.

## 3 Investigation/Treatment

**FIGURE 1:**
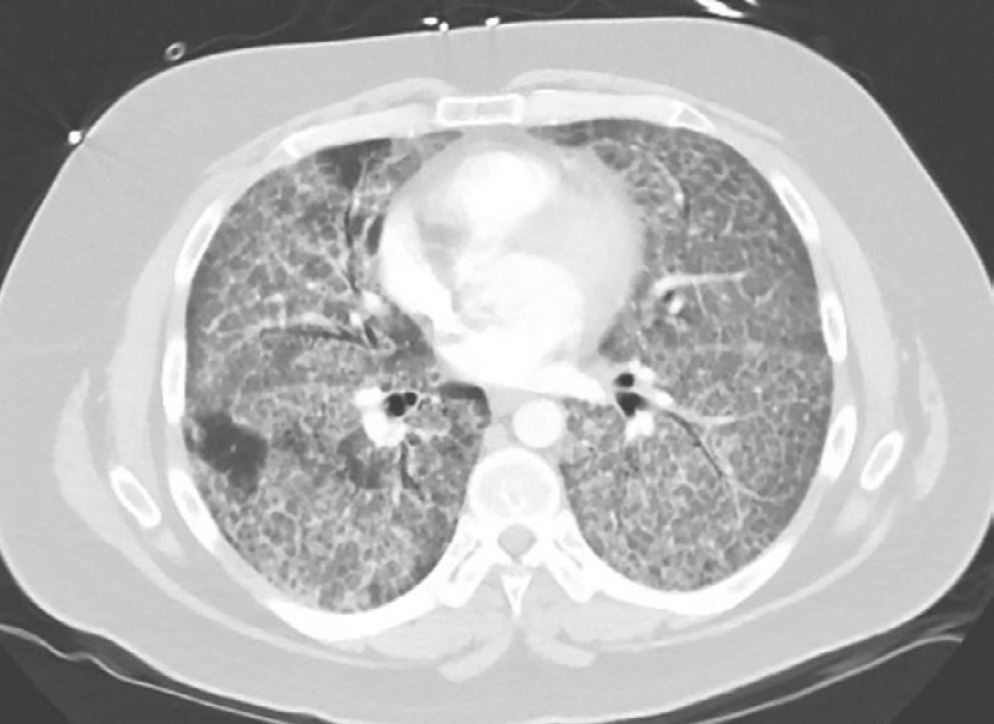
CT scan of the lungs showing widespread ground‐glass opacities and thickened septal lines in all five lobes, described as ‘crazy paving pattern’.

Chest x‐ray showed diffuse, bilateral parenchymal opacities. CT chest showed widespread ground‐glass opacities, thickened septal lines, and a ‘crazy paving’ pattern (Figure 1). Pulmonary function tests (PFTs) were done which revealed forced vital capacity of 2.52 L (43% predicted), FEV1 of 2.18 L (48% predicted) with a normal FEV1/FVC ratio. Total lung capacity was reduced to 4.81 L (61% predicted) with severe reduction in DLCO (24% predicted). Bronchoalveolar lavage (BAL) and lung biopsy revealed alveoli filled with periodic acid–Schiff (PAS) positive material confirming a diagnosis of PAP. He underwent two whole lung lavages (WLL) within a month and was gradually weaned off oxygen. A follow‐up CT scan after 2 weeks showed improvement in his radiographic findings. In particular, the crazy paving pattern was almost resolved, although there was some suggestion of mild alveolitis. He underwent a 6‐min walk test and had good work capacity without any desaturation. After a year, he started developing dyspnea again and a CT scan of the chest revealed progressive lung opacities consistent with PAP. Anti‐granulocyte macrophage colony stimulating factor antibodies (anti‐GM‐CSF) titers were sent at the time which were increased. He required five more WLLs in the next 3 months with no improvement. He was started on inhaled 250 mg twice daily of GM‐CSF, but he had only mild improvement in symptoms. He completed six cycles of plasmapheresis and did well; however, after 3 months his symptoms recurred and his oxygen requirement went up to 8 L NC at rest. A trial of Rituximab was then attempted and he was given 1 g IV Rituximab for 2 doses, 2 weeks apart. He experienced a partial response for a few months, but afterwards continued to have increasing oxygen requirements, needing two more WLLs in 2 months. By this time, he had 15 WLLs in almost 3 years and recurrent mycobacterium avium complex infections for which he received rifampin, ethambutol, and azithromycin resulting in clearance. As all standard treatment options had failed to control the disease, it was then decided to try chemotherapeutic agents to suppress the production of autoantibodies. He was started on weekly cyclophosphamide, bortezomib, and dexamethasone (CyBorD), a regimen used as induction therapy for multiple myeloma. With CyBorD, symptoms improved, which correlated with decreasing anti‐GM‐CSF titers, but after 4 cycles, further coverage was denied by insurance. He then started on 1 g Rituximab every 2 months and 500 mg inhaled GM‐CSF twice daily.

## 4 Outcome and Follow‐Up

**FIGURE 2:**
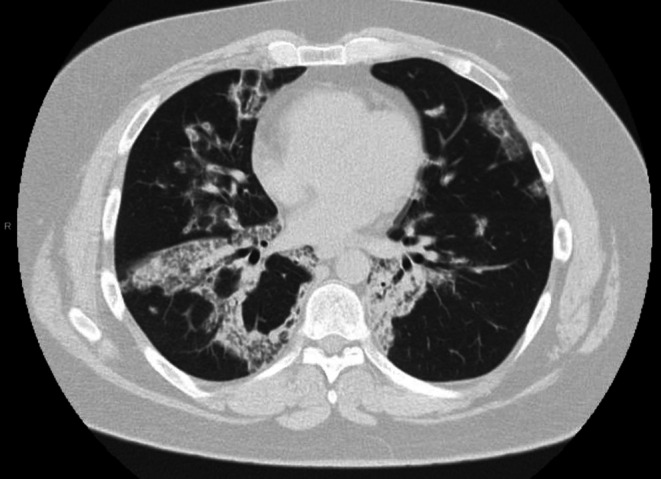
CT scan showing decreasing burden of pulmonary alveolar proteinosis with significantly improved clearance of the bilateral lower lobes.

He had significant improvement in symptoms with regular Rituximab doses and decreasing anti‐GM‐CSF titers. CT scan showed decreasing burden of pulmonary alveolar proteinosis with improved clearance of the bilateral lobes (Figure 2). Gradually he was switched to 12‐weekly 1 g Rituximab dosing and was weaned completely off oxygen. We tried to increase the interval between infusions to 4 months; however, due to recurrence of symptoms, we continued the 3‐monthly regimen. He continues to be followed in our clinic.

## 5 Discussion

In patients with PAP, CT scan shows ground glass appearance of alveoli and thickened septa, a pattern referred to as crazy paving pattern [5]. It is pathognomonic of PAP but may be seen in a variety of diseases like acute respiratory distress syndrome, bronchioloalveolar carcinoma, alveolar hemorrhage, pulmonary edema, interstitial pneumonitis, or various types of pneumonias [6]. A significant reduction in diffusion capacity and an increase in the alveolar‐arterial gradient are the most common findings on PFTs. Bronchoscopy with bronchoalveolar lavage is the gold standard for diagnosis, which reveals cloudy, milk‐like fluid, and large foamy macrophages with amorphous material that stain positive for PAS [3]. This along with typical radiographic findings on CT scan confirms a diagnosis of PAP without the need to undergo more invasive testing. GM CSF antibody testing should be done in all patients to help determine the type of PAP. Lung biopsy should only be pursued if the above testing is negative as it is invasive and may be equivocal due to patchy involvement of disease [5, 7].

WLL is standard treatment for PAP, which involves intubating the patient and inserting warm normal saline through the endotracheal tube, washing one lung at a time. There is mixed data regarding the efficacy of WLL. It has been shown to improve pulmonary function and reduce symptoms over time [5]. In our patient despite repeated WLLs, the response was not sustained, needing us to explore other options. In patients with autoimmune PAP, inhaled GM‐CSF should be used which delivers GM‐CSF directly into the alveoli [5]. For refractory disease, plasmapheresis and rituximab are used which aim to target the circulating GM‐CSF antibodies. The dosing of Rituximab used in prior studies has been 1000 mg twice a month. It improves oxygen, decreases anti GM‐CSF titers, and decreases the frequency of WLLs [8]. Rituximab maintenance therapy is not standardized, and frequency of administration differs [5, 9, 10]. We decided to continue our patient on regular infusions due to recurrence of symptoms. Patients who do not respond to Rituximab can be trialed on plasmapheresis. Prior case reports have shown mixed response with only a few cases demonstrating sustained improvement in symptoms. [5] Our patient did not benefit from plasmapheresis and was symptom free only for 3 months afterwards. Systemic corticosteroids have previously been trialed in autoimmune PAP but are ineffective and increase the risk of pulmonary infections [11].

CyBorD is plasma‐cell directed chemotherapy, which is used for multiple myeloma and has shown efficacy in other life‐threatening autoimmune diseases [12, 13, 14]. Various chemotherapy drugs have previously been used to treat autoimmune PAP when it coexists with myeloproliferative malignancies [15, 16, 17, 18]. However, autoimmune PAP alone without a coexisting malignancy has previously not been treated with the chemotherapy regimen that was used in our case. Further research is needed to determine the effectiveness, safety, and long‐term outcomes related to the use of chemotherapy in patients with autoimmune PAP. A recent case report describes daratumumab, a CD38‐directed therapy, as a novel treatment with benefit in refractory autoimmune pulmonary alveolar proteinosis through depletion of anti–GM‐CSF–producing plasma cells [19]. Severe PAP can lead to irreversible lung damage requiring lung transplantation, which can be tried for patients with refractory disease who fulfill the International Society for Heart and Lung Transplantation (ISHLT) criteria for patients with interstitial lung disease [20]. A recent Retrospective International Multicenter Analysis that gathered lung transplantation data for PAP from 162 centers between 1990 and 2023 demonstrated survival rates of 90.8% at 1 year, 85.1% at 3 years, and 69.9% at 5 years [21].

Response to therapy can be assessed by symptomatic improvement as well as progress on the 6‐min walk test [22]. It has been shown that CT scan features before and after treatment also correlate well with PaO2 (arterial oxygen pressure) [23]. Furthermore, changes noted on serial CT scans act as an important predictor of clinical improvement and prognosis of the disease [24]. Decreasing anti GM‐CSF titers may indicate improvement in disease burden. KL‐6 (Krebs von den Lungren protein‐6) has been proven useful in assessing progression of the disease and the need for therapy as well [25]. In our patient, treatment was guided based on symptoms, imaging, and anti GM‐CSF titers.

This case highlights treatment options for patients with autoimmune PAP, particularly in those patients who do not respond to conventional therapy. More studies are required to establish the efficacy and long‐term outcomes for Rituximab and chemotherapy agents used for autoimmune PAP.

## Author Contributions

**Meher Binte Ali:** conceptualization, investigation, writing – original draft, writing – review and editing. **Danish Jilani:** writing – original draft, writing – review and editing. **Syeda Hafsa Qadri:** writing – original draft, writing – review and editing. **Yilin Song:** writing – original draft, writing – review and editing. **Nancy Hardy:** conceptualization, formal analysis, supervision, validation, writing – original draft, writing – review and editing.

## Funding

The authors have nothing to report.

## Ethics Statement

The authors have nothing to report.

## Consent

Written informed consent was obtained in accordance with the journal's patient consent policy.

## Conflicts of Interest

The authors declare no conflicts of interest.

## Data Availability

Data sharing not applicable to this article as no datasets were generated or analysed during the current study.

## References

[1] C. McCarthy , R. Avetisyan , B. C. Carey , C. Chalk , and B. C. Trapnell , “Prevalence and Healthcare Burden of Pulmonary Alveolar Proteinosis,” Orphanet Journal of Rare Diseases 13 (2018): 129. 30064481 10.1186/s13023-018-0846-y PMC6069872

[2] L. B. Jehn and F. Bonella , “Pulmonary Alveolar Proteinosis—Current and Future Therapeutical Strategies,” Current Opinion in Pulmonary Medicine 29, no. 6 (2023): 450–458. 10.1097/MCP.0000000000000982 37395514

[3] A. Kumar , B. Abdelmalak , Y. Inoue , and D. A. Culver , “Pulmonary Alveolar Proteinosis in Adults: Pathophysiology and Clinical Approach,” Lancet Respiratory Medicine 9, no. 10 (2021): 1077–1088. 29397349 10.1016/S2213-2600(18)30043-2

[4] C. McCarthy , M. Kokosi , and F. Bonella , “Shaping the Future of an Ultra‐Rare Disease: Unmet Needs in the Diagnosis and Treatment of Pulmonary Alveolar Proteinosis,” Current Opinion in Pulmonary Medicine 28, no. 5 (2022): 401–410. 10.1097/MCP.0000000000000601 31365379

[5] C. McCarthy , F. Bonella , M. O'Callaghan , et al., “European Respiratory Society Guidelines for the Diagnosis and Management of Pulmonary Alveolar Proteinosis,” European Respiratory Journal 65, no. 1 (2025): 230–241. 10.1183/13993003.00725-2024 39147411

[6] Y. Feng , J. Zhao , Q. Yang , et al., “Pulmonary Melanoma and “Crazy Paving” Patterns in Chest Images: A Case Report and Literature Review,” Medicine (Baltimore) 97, no. 34 (2018): e12010. 27488496 10.1186/s12885-016-2630-5 PMC4973081

[7] C. McCarthy , B. Carey , and B. C. Trapnell , “Blood Testing for Differential Diagnosis of Pulmonary Alveolar Proteinosis Syndrome,” Chest 155, no. 3 (2019): 450–452. 10.1016/j.chest.2018.11.002 PMC6688977 30732696

[8] M. S. Kavuru , A. Malur , I. Marshall , et al., “An Open‐Label Trial of Rituximab Therapy in Pulmonary Alveolar Proteinosis,” New England Journal of Medicine 361, no. 21 (2009): 2183–2194. 21478218 10.1183/09031936.00197710 PMC3874725

[9] D. Bird , J. Evans , and C. Pahoff , “Rituximab Rescue Therapy for Autoimmune Pulmonary Alveolar Proteinosis,” American Journal of Respiratory and Critical Care Medicine 181, no. 8 (2010): 1025–1027. 10.1016/j.rmcr.2022.101637 PMC8943437 35342706

[10] A. Amital , S. Dux , D. Shitrit , et al., “Therapeutic Effectiveness of Rituximab in a Patient with Unresponsive Autoimmune Pulmonary Alveolar Proteinosis,” Chest 142, no. 2 (2012): 513–515. 20855439 10.1136/thx.2010.140673

[11] K. Akasaka , T. Tanaka , N. Kitamura , et al., “Outcome of Corticosteroid Administration in Autoimmune Pulmonary Alveolar Proteinosis: A Retrospective Cohort Study,” BMC Pulmonary Medicine 19 (2019): 68. 26264717 10.1186/s12890-015-0085-0 PMC4534060

[12] C. B. Reeder , D. E. Reece , V. Kukreti , et al., “Cyclophosphamide, Bortezomib, and Dexamethasone Induction for Newly Diagnosed Multiple Myeloma: High Response Rates in a Phase II Clinical Trial,” Leukemia 23, no. 7 (2009): 1337–1341. 19225538 10.1038/leu.2009.26 PMC2711213

[13] Y. Kusne , R. Fonseca , M. Arribas , et al., “Cyclophosphamide‐Bortezomib‐Dexamethasone (CyBorD) ± Daratumumab Treatment Results in Sustained Hematologic Response in Patients With AL Amyloidosis: A Retrospective Analysis at Mayo Clinic,” Blood 140, no. Supp 1 (2022): 7224–7225.

[14] R. Pasquale , J. A. Giannotta , W. Barcellini , and B. Fattizzo , “Bortezomib in Autoimmune Hemolytic Anemia and Beyond,” Therapeutic Advances in Hematology 12 (2021): 20406207211046428. 34795889 10.1177/20406207211046428 PMC8593301

[15] C. P. Chaulagain , M. Pilichowska , L. Brinckerhoff , M. Tabba , and J. K. Erban , “Secondary Pulmonary Alveolar Proteinosis in Hematologic Malignancies,” Hematology/Oncology and Stem Cell Therapy 7, no. 4 (2014): 127–135. 25300566 10.1016/j.hemonc.2014.09.003

[16] N. Imoto , N. Harunori , K. Furukawa , et al., “GM‐CSF Autoantibody‐Positive Pulmonary Alveolar Proteinosis With Simultaneous Myeloproliferative Neoplasm,” Internal Medicine 56, no. 4 (2017): 435–439. 28202867 10.2169/internalmedicine.56.6920 PMC5364198

[17] K. Ohmachi , D. Ogiya , F. Morita , et al., “Secondary Pulmonary Alveolar Proteinosis in a Patient With Chronic Myeloid Leukemia in the Accelerated Phase,” Tokai Journal of Experimental and Clinical Medicine 33, no. 4 (2008): 146–149. 21318986

[18] M. Yoshimura , K. Kojima , R. Tomimasu , et al., “ABL Tyrosine Kinase Inhibitor‐Induced Pulmonary Alveolar Proteinosis in Chronic Myeloid Leukemia,” International Journal of Hematology 100 (2014): 611–614. 25212679 10.1007/s12185-014-1666-z

[19] A. Strong , Y. Sun , D. Pilcher , Z. Kaplan , and R. G. Stirling , “The Novel Use of Daratumumab in the Treatment of Refractory Autoimmune Pulmonary Alveolar Proteinosis,” Respirology Case Reports 13, no. 6 (2025): e70246. 40535728 10.1002/rcr2.70246 PMC12174958

[20] M. M. Crespo , E. D. Lease , A. Sole , et al., “ISHLT Consensus Document on Lung Transplantation in Patients With Connective Tissue Disease: Part I,” Journal of Heart and Lung Transplantation 40, no. 11 (2021): 1251–1266. 10.1016/j.healun.2021.07.014 34417111

[21] S. Schwarz , H. Prosch , E. Yekeler , et al., “Lung Transplantation for Pulmonary Alveolar Proteinosis: A Retrospective International Multicenter Analysis,” Journal of Heart and Lung Transplantation 44, no. 4 (2025): S126.

[22] C. McCarthy , B. C. Carey , and B. C. Trapnell , “Autoimmune Pulmonary Alveolar Proteinosis,” American Journal of Respiratory and Critical Care Medicine 205, no. 9 (2022): 1016–1035. 35227171 10.1164/rccm.202112-2742SO PMC9851473

[23] S. Tokura , M. Akira , T. Okuma , et al., “A Semiquantitative CT Grading System for Evaluating Therapeutic Response in Pulmonary Alveolar Proteinosis,” Annals of the American Thoracic Society 14, no. 9 (2017): 1403–1411. 28489417 10.1513/AnnalsATS.201607-574OC

[24] B. Da Nam , T. J. Kim , M. P. Chung , et al., “CT Findings in Pulmonary Alveolar Proteinosis: Serial Changes and Prognostic Implications,” Journal of Thoracic Disease 10, no. 10 (2018): 5774–5781. 30505485 10.21037/jtd.2018.09.86 PMC6236152

[25] F. Bonella , S. Ohshimo , C. Miaotian , M. Griese , J. Guzman , and U. Costabel , “Serum KL‐6 Is a Predictor of Outcome in Pulmonary Alveolar Proteinosis,” Orphanet Journal of Rare Diseases 8 (2013): 53. 23557396 10.1186/1750-1172-8-53 PMC3629718

